# The Exploitation of Data from Remote and Human Sensors for Environment Monitoring in the SMAT Project

**DOI:** 10.3390/s121217504

**Published:** 2012-12-17

**Authors:** Rosa Meo, Elena Roglia, Andrea Bottino

**Affiliations:** 1 Department of Computer Science, University of Torino, C.so Svizzera 185, 10149 Torino, Italy; 2 European Commission, Joint Research Centre, Institute for Environment and Sustainability, Via E. Fermi 2749, 21027 Ispra (VA), Italy; E-Mail: elena.roglia@jrc.ec.europa.eu; 3 Department of Control and Computer Engineering, Polytechnic of Torino, C.so Duca degli Abruzzi 24, 10129 Torino, Italy; E-Mail: andrea.bottino@polito.it

**Keywords:** mosaicking, orthoframes, volunteered geographic information, cartography, metadata

## Abstract

In this paper, we outline the functionalities of a system that integrates and controls a fleet of Unmanned Aircraft Vehicles (UAVs). UAVs have a set of payload sensors employed for territorial surveillance, whose outputs are stored in the system and analysed by the data exploitation functions at different levels. In particular, we detail the second level data exploitation function whose aim is to improve the sensors data interpretation in the post-mission activities. It is concerned with the mosaicking of the aerial images and the cartography enrichment by human sensors—the social media users. We also describe the software architecture for the development of a mash-up (the integration of information and functionalities coming from the Web) and the possibility of using human sensors in the monitoring of the territory, a field in which, traditionally, the involved sensors were only the hardware ones.

## 1. Introduction

The main challenges for the safety of the citizens and the environment vary from organized crime to situations caused by political and economic instability, from the security of critical infrastructures for the preservation of artistic and cultural heritage to the effects of climatic change, from the energetic safety to the monitoring and surveillance of the territory to prevent environmental disasters or to effectively intervene when these occur. For these reasons, in recent years both the European Union (UE) and the local authorities have funded different research projects on the use of advanced technologies for territory monitoring and surveillance.

The technologies related to UAVs are currently one of the most growing research areas for surveillance missions using remote sensing tools such as infrared sensors, Synthetic Aperture Radar (SAR) or electro-optical sensors. This is due to different attractive factors. One of them is certainly the absence of risks for the human pilot involved in dangerous operations like fire or avalanche monitoring during bad weather conditions. In addition, UAVs can be used to fly at low altitudes, producing in this way very high resolution imagery (compared to satellite imagery), often in near real time. This feature is greatly appreciated for land use monitoring missions and crops control in selected areas. Moreover UAVs can be tailored to particular tasks. For example, if equipped with electro-chemical sensors, they can be used for pollution emissions control.

UAVs can be deployed both in the so-called “DDD” (Dull, Dirty, and Dangerous) missions and in routine missions, requiring a long flight endurance, such as ordinary and extraordinary land monitoring. Furthermore, life-cycle costs of Unmanned Air Systems (UAS) are lower than those of comparable manned aircrafts [1].

SMAT-F1 (Sistema di Monitoraggio Avanzato del Territorio-Fase 1) (Advanced Monitoring System of the Territory-Phase 1) has been the first phase of a research project funded by the Piedmont Region (Italy) and co-funded by the European Commission for the regional development of the advanced territory monitoring.

The project, started in January 2009 and ended in March 2012, was aimed at:

- defining the requirements for a UAVs-based surveillance system;
- designing and developing the Supervision and Control Station (SS&C) involved in the exchange of information between UAS and human operators and capable of supporting data processing;
- integrating the control stations (CS) of each UAV with the SS&C;
- demonstrating the operativeness of the entire system on a representative scenario.

The system has been planned to cover different potential needs in the Piedmont Region, such as the surveillance of areas subject to natural disasters or to human intervention (e.g., traffic, urban planning, pollution and cultivation) and the monitoring of specific areas for prevention and planning purposes. Furthermore, the system has been designed to deliver in real time information to authorities responsible of managing critical events.

The specific subject of this paper is the description of the Second Level Exploitation function of the SMAT-F1 system. The purpose of this function is the post-processing of the sensors data and their assessment from the viewpoint of the end-users. Its peculiarity is to combine traditional sensors with a novel generation of sensors, the human sensors, in order to enhance the mission outcomes with the deep and specific knowledge of the territory provided by them. The exploitation of human sensors is enabled by the diffusion of those interactive and collaborative practices known as Web 2.0. Users of social networking sites, blogs and wiki contribute to provide valuable information concerning the territory, the multiform human activities carried out in the environment and the natural resources exploitation. In the SMAT-F1 project, this information, labelled with its geo-position and temporal validity, is filtered by statistical validation procedures and then used to annotate the traditional cartography, which is finally made interactive and superimposed to the aerial images acquired by the image sensors.

The rest of the paper is organized as follows. Section 2 describes an overview of the overall architecture of the SMAT-F1 system. Section 3 details the software architecture of the main component of the ground system. The description of the sensor data processing and integration is described in Section 4, together with an overview of related works. Section 5 describes more in particular the process-flow and the algorithms employed for the aerial image processing. Section 6 describes how human sensors are exploited for the cartography enrichment function and how the information fusion (referred to as a mash-up) is built. Finally, experiments and results are presented in Section 7, while Section 8 draws the conclusions and sketches future works.

## 2. The Overall Architecture of the SMAT-F1 System

Three main segments compose the SMAT-F1 architecture:

- aerial segment
- ground segment
- communication segment

**Figure 1.**
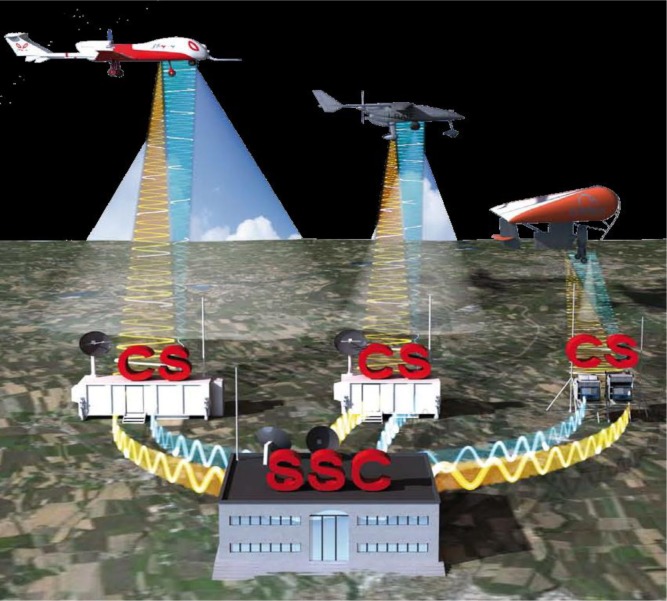
The main components of the overall SMAT-F1 system. Reproduced with permission from the SMAT project consortium, SMAT-F1 brochure; published online. See [2].

As shown in Figure 1, the aerial segment is constituted by three different UAV, equipped with proper remote sensors (e.g., optical sensor, infra-red sensor and SAR) and operating at different altitudes and at different speeds according to the mission requirements.

Each UAV has a Control Station (CS) responsible for driving the flight of the aircraft, controlling the use of the payload sensors and receiving and distributing the information collected during the flight. The three CS and the SS&C make up the ground segment. The SS&C is connected to each CS and is responsible of coordinating the activities of the UAS and processing the information arriving from the different platforms.

The communication segment is made up of two types of links: those between each CS and its UAV and those between each CS and the SS&C.

The SS&C is the heart of the system architecture. It is involved in the integrated monitoring of sensors data, mission planning support, data processing and data fusion. Furthermore, it supports its operators in managing and controlling the mission plans of multiple UAVs, making them coexist and collaborate. This task is particularly complex, since the operative modes of the different UAV platforms are independent and autonomous, each having its own proprietary implementation choices, such as those concerning flight control mode, sensor configuration, communication and data storage protocols and so on.

Other functions of the SS&C are handling data storage and persistence, and managing geo-spatial and temporal referencing of the collected information. Additionally, the system must post-process the archived data, a functionality referenced in the system as Second Level Data Exploitation whose main tasks are the following. First, create a photogrammetric covering of the mission area, mosaicking the images acquired from the UAVs, which is then geo-referenced and superimposed with the territory cartography. Second, enhance the information content of the data and of the cartography, in order to improve their usefulness for the end-users. This is achieved integrating them with geo-referenced users-generated contents, obtained from external sources as, for instance, social media or Web applications (like OpenStreetMap, which we will describe hereafter). As a matter of facts, the human tagging activity on the territory is nowadays so ubiquitous and rich to allow speaking of human sensors, where this term summarizes the availability of a large amount of timely updated, geo-referenced annotations created by users and available through the Web. Since this information is provided voluntarily by individuals in open, collaborative projects, it is referenced as well as Volunteered Geographic Information (VGI).

## 3. The Software Architecture for the SS&C and the Second Level Exploitation of Data

The software architecture developed for the SMAT-F1 project is a high performance, broad bandwidth service oriented architecture that supports coupling of archives and real-time geo-spatial data with scientific applications such as simulation, visualization and analytical software [3]. The system architecture is the result of working activities involved in SMAT-F1. Many of the system features were developed by project partners. For this reason and for the project policies, system components are described only at a high-level of detail.

### 3.1. The Software Architecture Developed for the SS&C

The software architecture is designed as a three-tier system: a client, a middle-ware and a data layer. Each layer has the role to control a specific category of software components: database services, geo-processing services and user applications.

The data storage layer answers requests from other layers, providing the data persistence and offering services for the archival, the retrieval and the management of all the data handled in the SS&C (e.g., mission data, simulation and historical data). The services it offers act as a neutral data interface for the exchange of information between the components of the SS&C: all the system functions perform their activity taking their input information from the data storage level and returning their output in the same storage level. The multiplicity of data involved in the system requires, for their management, the integration of both a relational DBMS (PostgreSQL), for structured data, and a GIS (POSTGIS), for geographical data. Large textual documents, sensor images and videos are stored on the file system.

The middle-ware tier provides different computational services. Some of them are related to tactical planning, mission execution, planning simulation, real time monitoring, Second Level Exploitation and data assessment. Other functions are designed to handle data exchanged with external devices (data acquisition, data distribution and data visualization functions) and to interact with the Communication Channel. In the context of the SMAT project this software layer is executed and managed in the SS&C.

The system GUI is Web-based and provides a range of functionalities including querying the archives, displaying results (e.g., metadata and maps), performing spatial visualization or system administration.

**Figure 2.**
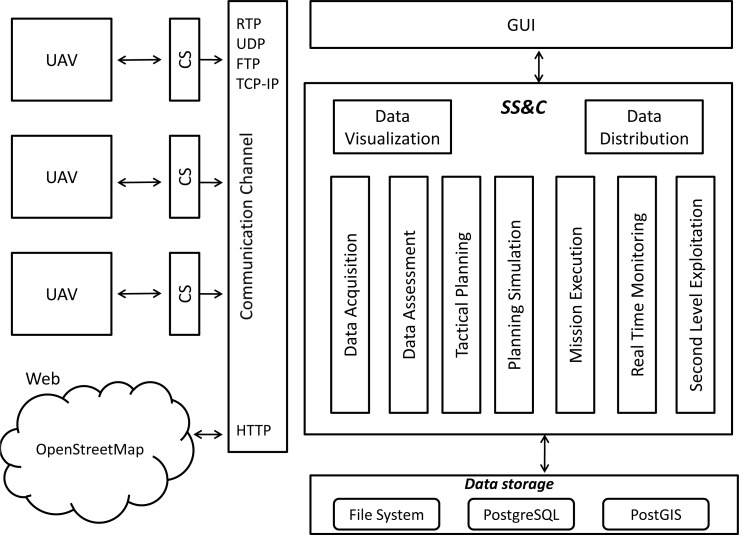
SMAT-F1 software architecture.

Figure 2 shows the main components involved in the architecture.

Different functionalities are available in the SS&C. The Data Assessment function allows performing detailed post mission analysis and mission report generation. The Tactical Planning function supports SS&C users during the development of the mission plan, the overall strategic plan of multiple UAS working together. The Planning Simulation Function can be used to simulate the mission plans. The Mission execution function handles all the necessary data to perform Data Visualization and real-time mission monitoring, requesting them to the data storage layer. The Real Time Monitoring provides computational capabilities as well, *i.e*., algorithms used to process the received data, and generate additional information for the users benefit. The Second Level Exploitation function provides the functionalities for data comparison and correlation, data conversion, data representation, Digital Terrain Model (DTM) generation and annotation. The Data Distribution Function manages the data collection (*i.e*., mission major events, failures and problems, operational area description, mission plan data, recorded video and images, *etc*.) and the reports generation. The Data Visualization services provide to the operator several functionalities related to Real Time Mission Monitoring, Post Mission Data Processing and Mission Planning.

The Communication Channel is used to manage the interchange of information between the CSs and SS&C and between SS&C and the Web. In particular, telemetry data is transmitted in synchronous mode from the CSs to the SS&C through a TCP/IP port that communicates with a Telemetry Acquisition Functions. Files are exchanged in asynchronous mode between CSs and SS&C using a FTP manager. Video is transmitted in synchronous mode using a RTP/UDP port that communicates with a Video Data acquisition function. All data collected are sent to the data storage functions. In addition the Video Data acquisition function sends data to the Real Time Monitoring function as well.

The next section describes in greater detail the Second Level Exploitation services. For a complete description of the implemented system functionalities, the interested reader is referred to [3].

### 3.2. The Second Level Exploitation

The Second Level Data Exploitation has been defined as a post-mission activity that exploits the richness of information gathered from different sources (video, telemetry, images, *etc*.) and allows the generation of information that could be useful in defining new mission plans. Analyses of mission data often requires comparison and correlation with historical data and, in case of necessity, all Second Level Exploitation algorithms allow data re-processing under operator request in order to extract necessary and missing information from actual or past missions.

The Second Level Exploitation involves different operations like image processing, orthorectification, image mosaicking, data comparison and correlation, data representation, data annotation with external sources and, more generally, data enrichment.

This function is aimed at adding metadata as annotations to geo-referenced data stored in the SS&C Data Model (e.g., targets, waypoints, flown points, airports). The annotations are provided to the SS&C operator in the form of suggestions that could become persistent if they meet operator needs. They are supplied through a mechanism that collects and merges metadata extracting information both from the historical (geo-referenced) stored data and through external sources, such as Web applications. In particular, the work proposed in this paper shows how it is possible to harvest data from Web 2.0 applications and social networks.

## 4. Overview of the Activity on Sensors in SMAT-F1 and Related Works

This section provides an overview of the activities performed on the sensors data in the SMAT-F1 project and summarizes the related works.

- Recently the availability of aerial images increased substantially, and algorithms for their automatic processing are sorely needed. Many applications, such as surveillance, monitoring, line inspection, disaster and emergency management and others, require to generate a photogrammetric coverage of an extended area [4,5]. This can be obtained assembling video streams or image sequences taken from on-board cameras into an *image mosaic*, which is a wide single image covering all the region of interest. The computed mosaic can then be used, for instance, to develop Google Maps like applications to let users inspect details by panning and zooming the mosaic, and also to overlay other available geo-referenced data (cartography, images and so on). The mosaic should have two properties. First, it must be consistent with some geodetic reference system so that it is possible to carry out measurements on it and to match it to other photogrammetric coverages or to cartographies of the same area. Second, the final merge should be seamless and not blurred. This requires a correct choice of re-sampling scaling factors (in order to not over-sample the original data), a precise alignment of overlapping frames and the use of convenient frame blending techniques. Precise automatic mosaicking is still a big challenge, while standard techniques often need manual intervention to match frames and reference cartography through individuation of Ground Control Points (GCP). In this work we present an approach to fully automate the mosaicking process. Frames are first orthorectified and geo-referenced according to on-board sensor data, then their alignment is refined through their own visual content. Finally all frames are merged into the overall mosaic.
- Citizen as sensor for data enrichment function: on the Web there exists a large amount of geographical information provided by persons that use social networks adding user-contributed and location-related contents. Produced data are very often free and constantly updated. For these reasons we decided to exploit the richness of information available on the Web and to integrate this information with the traditional maps, which are costly and get very fast outdated. Furthermore, cartographic maps are often thematic and do not contain all the information needed by the user. On the contrary, people can provide geographical information to the social networks through handhelds, pocket PCs or mobile phones connected to Internet while moving around the territory. They act in this way as human sensors that collect information from their local environment and perform processing and analysis of the collected data. This pre-processing/processing of sensory data from experience/background knowledge is what differentiates our sensing capabilities from hardware sensors. In citizen sensing, a sensor is not necessarily a hardware sensor but can be a virtual sensor or a human interpreting sensory data [6].
- The information gathered by the human sensors can be integrated with the information collected by traditional methods and can be useful to increase the information level of professionals and decision makers especially for the environmental monitoring and for the prevention of emergencies. In Section 6 we describe how we performed data enrichment using this type of data.

### 4.1. Existing Projects Involving UAVs

A fleet of different UAVs has many potentialities for natural disasters surveillance. They can be used with proficiency in disaster assessment, response and management [7]. The feasibility of using a fleet of multiple low-altitude, short endurance (LASE) UAVs to cooperatively monitor and track the propagation of large forest fires has been presented in [8]. A framework for cooperative fire detection by means of a fleet of heterogeneous UAVs has been presented in [9]. The authors of [10] described COMETS (Real-Time Coordination and Control of Multiple Heterogeneous Unmanned Aerial Vehicles), a project aimed at designing a system for cooperative activities using heterogeneous UAVs involved in fire detection and monitoring and in terrain mapping. A fleet of UAVs has also been studied as a low-cost remote sensing system for the high-resolution acquisitions of landslides [11], in operational oil spill surveillance, monitoring and assessment [12] and to capture building damage resulting from hurricane events [13]. In agriculture, multiple UAVs have also been used for surveying the river environment [14], for the acquisition of imagery of crops and assessment of vegetation indexes relevant to the vegetation analyses [15] and for the rangeland management [16]. The wide application area for UAV includes also very variegate monitoring activities such for example, the monitoring of freeway conditions, track vehicle movements and parking lot utilization [17], the monitoring of the gas pipelines [18] and of the archaeological excavations [19].

### 4.2. Aerial Image Mosaicking: Related Works

Orthorectification and mosaicking are among the most relevant outcomes of UAV image processing. They allow building a single seamless wide image, covering the large areas to be monitored, which can be geo-referenced and integrated with other geo-spatial data or compared with previous aerial images of the same area. Many approaches to the problem have been presented in literature. Several works are based on GCPs, whose known positions on the terrain are matched with their coordinates in the UAV images in order to compute the transformation matrices mapping one image to the other [20–22]. However, this technique often requires user interaction for the accurate identification of GCPs on images, or it cannot be applied in areas where disruptive events, like flood fills or landslides, occurred. Nowadays, the increasing availability of high quality Global Positioning System (GPS) and Inertial Measurement Unit (IMU) sensors allows deriving position and orientation of the camera to geo-reference image data with an accuracy adequate for several applications. Despite that, the absolute precision of these sensors is not yet sufficient to obtain seamless mosaicking. To overcome this drawback, image metadata (GPS/IMU data) have been often coupled with computer vision (CV) techniques in order to exploit visual coherence too in the image matching process. A survey of CV techniques for image mosaicking can be found in [23]. The general structure of vision based UAV mosaicking approaches is the following. First, images are globally aligned according to their metadata and salient features are identified in each image. Then, correspondences between features of neighboring images are established, usually by matching some kind of feature descriptors, and, from these correspondences, a mapping between the images is computed. Finally, transformed images are blended to obtain the final mosaic. In [24], the initial image alignment given by the telemetry is corrected using a pyramid-based matching algorithm. However, image orthorectification is only approximated, using a pseudo parallel projection, in order to reduce the computational burden. The same problem affects the approach presented in [25], where the ground is considered as a planar surface. Then, homographies relating two images are computed from simple feature descriptors, based on image patches centered at corners identified in the image. In this process, each image is represented by a “bag-of-words” (BoW), the set of its descriptors, and different BoWs are compared for image matching. The algorithm described in [26] quickly creates an initial mosaic of pseudo orthogonal images that is further refined, as more images are available over time, by optimizing a quality function, which involves visual constraints, in a short neighborhood of the camera position computed from UAV sensors. In order to produce orthomosaics, free of distortion also in areas with elevation variations and suitable for area or distance measurements, several approaches (as [20,22,27–29]) take into account a correct image orthorectification. It should be underlined that a possible drawback of hybrid approaches, which base image registration on both metadata and visual contents, is that image alignment is often an iterative approach, where each new image to be added to the mosaic is matched with a previously aligned image. As a result, the concatenation of pairwise image transformations can lead to accumulated errors. In order to solve this problem, several works (as [22,30,31]) proposed to use a bundle adjustment, which simultaneously refines all the transformation parameters according to an optimality criterion based again on the correspondences between corresponding features in the different images.

### 4.3. Elaboration and Analysis of Human Sensors and VGI: Existing Projects and Related Works

Several works deal with the integration of VGI data with geographical data collected with traditional methods. In recent years, different projects have used VGI to facilitate crisis mapping in area hit by disasters. After the 2010 Haiti earthquake, the OpenStreetMap community started to enter data in the OpenStreetMap database as soon as the aerial photos of the areas affected by the earthquake were made available. Within a few hours, it had been possible to generate updated maps of island, showing the roads still passable, the positions of the refugee camps, the bridges unused and additional information useful for rescue operations. Ushahidi [32] is an open source platform that enables the easy deployment of crowd-sourced interactive mapping applications with (SMS) text messages, email or Web and visualize it on a map or time-line. Initially, it has been developed for monitoring post-election violence following the Kenyan presidential election of 2007, recently it has been used to crowd-source and map crisis information from multiple data streams in real-time during 2010 earthquake in Haiti and the Thailand flood of 2011.

VGI can be used in support to crisis management activities. During December 2010 to February 2011, the State of Queensland experienced a series of damaging floods that caused billions of dollars in damage and the loss of over 20 lives. At the peak of the Queensland floods there were between fourteen and sixteen thousand tweets per hour on the ‘#qldfloods’ hashtag, which was used to coordinate the conversation around the flood event itself. These peaked at around the time Brisbane and the surrounding areas began to become inundated. Agencies and organisations alongside members of the community began using the Twitter platform as a place to distribute ’raw’ footage and information, but then began to trust and ’follow’ particular accounts [33]. In May 2009 the Jesusita Fire in Santa Barbara, burned for 2 days and consumed 75 houses. Several individuals and groups immediately established volunteer map sites, synthesizing the VGI and official information that was appearing constantly. The officially reported perimeter of the fire was constantly updated based on reports by citizens. By the end of the emergency there were 27 of these volunteer maps online, the most popular of which had accumulated over 600,000 hits and had provided essential information about the location of the fire, evacuation orders, the locations of emergency shelters, and much other useful information [34].

The integration of human observation with sensor observation has been analyzed for building a noise mapping community in [35]. By integrating sensor observations measured by carried smart phones and by taking into account human observations the noise mapping capabilities of conventional sensor networks can be significantly enhanced. However, the accuracy of VGI remains a subject of considerable focus [36–38].

As regards the generation of the mash-up and the information integration process, we adopted the distributed solution for the software communication of the web services [39]. In order for the software parties to be able to inter-operate with each other, the service consumers must convert their local definitions of the information to the definitions of the service provider. Addressing these semantic concerns involves the usage of a common ontology as a mediation layer in order to abstract data terms, vocabularies and information into a shareable distributed model. Different works on semantics and geo-ontologies have focused on semantic interoperability. These works include the role of ontologies, for semantics-based and context aware retrieval of geographic information [40] and Semantic Geospatial Web services [39]. Ontologies are central to realizing the Semantic Web and Semantic Geospatial Web. W3C has adopted Resource Description Framework (RDF) [41] and Ontology Web Language (OWL) [42] as the standards for the representation of resources on the Web and semantic metadata. Vocabularies for data retrieval, information integration on the Web and a comprehensive ontology data-set are provided by the LinkedGeoData initiative [43], which is an effort to add a spatial dimension to the Web of Data/Semantic Web. LinkedGeoData uses the information collected by the OpenStreetMap project and makes it available as an RDF knowledge base according to the Linked Data principles.

## 5. The Algorithms for Aerial Image Mosaicking

**Figure 3.**
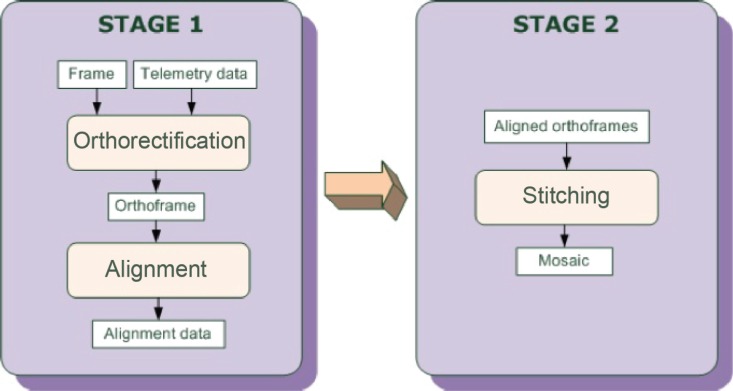
Overview of the Algorithm for Mosaicking.

The mosaicking algorithm is a two stage process, outlined in Figure 3. In the first stage, two main tasks take place:

- Orthorectification: each image is geometrically corrected according to its associated telemetry data, in order to convert its projection from perspective to orthographic and to remove distortions due to topographic relief and camera tilt;
- Alignment: an initial coarse alignment with a common reference system is obtained by geo-referencing each frame according to its telemetry information; this alignment is then refined by registering the rectified image with previously aligned orthoframes.

In the second stage of the mosaicking algorithm, aligned orthoframes are merged in a single image, with a suitable weighted blending, in order to get a seamless mosaic.

In the following subsections we will provide details about these tasks, as well as some results concerning the execution time of the proposed algorithm.

### 5.1. Orthorectification

UAV images are taken under a perspective projection, which does not provide metric information, since parallel lines converge in a vanishing point and angle and distances are not preserved. Therefore, in order to be useful for the described target applications, they must be orthorectified.

**Figure 4.**
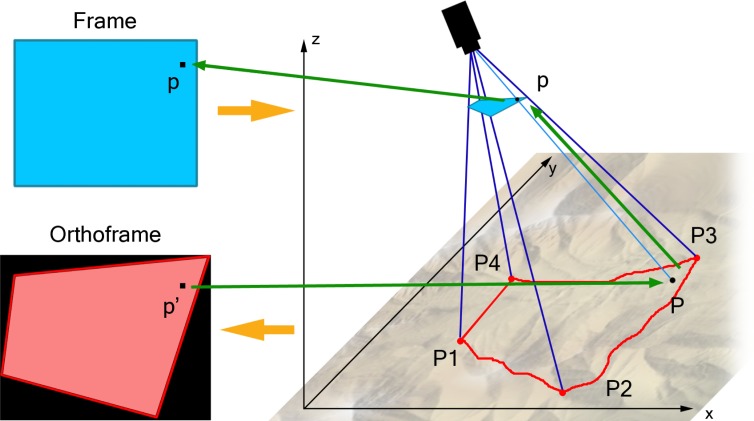
Orthorectification: value of each pixel *p′* is determined through a backtracking process to find the corresponding pixel *p* in the original frame.

The parameters of the orthographic projection can be computed from the ground positions *P*_1_–*P*_4_ of the points corresponding to the four image corners (see Figure 4) of the frame *F_i_*. The knowledge of the coordinates of these ground points allows as well to geo-reference *O_i_*, the final orthoframe.

Points *P_j_*, *j* = 1 . . . 4 are obtained as the intersection between the four rays *v_j_*, *j* = 1, . . . , 4, cast from the camera optical center and passing through the corner pixels of *F_i_*, and a Digital Elevation Model (DEM) of the observed area. Ray equations are computed from the projection matrix *M_i_*, evaluated through telemetry data associated with the *i^th^* frame (GPS position and camera altitude w.r.t. the chosen geodetic reference system). Since the DEM is represented as a sparse collection of points, *P_j_* coordinates are obtained using an iterative binary search over *v_j_* and a bilinear interpolation between neighbouring DEM points.

The computed orthographic projection matrix is then multiplied by a scaling factor *k*, in order to guarantee that the real world extension corresponding to a pixel is the same, on average, for *F_i_* and *O_i_*. The aim is to minimize both oversampling and down-sampling of *F_i_* while constructing *O_i_*.

The actual frame is orthorectified through a backtracking process. For each pixel *p′* of the ortho-image, its corresponding ground point *P* is computed inverting the orthographic projection and getting its elevation from the DEM. Then, *P* is projected on *F_i_* into *p* = *M_i_P* . Since *p* coordinates are not integers, the RGB values of *p′* are obtained as bilinear interpolation of the values of the four pixels of the original frame closer to *p*. If *p* falls outside *F_i_*, *p′* is left blank in *O_i_*.

Orthorectification is a parallelizable, per pixel, task. Therefore, in order to speed up its execution time, the algorithm has been implemented in GPU with the OpenCL programming language [44].

#### 5.1.1. Frame Filtering

**Figure 5.**
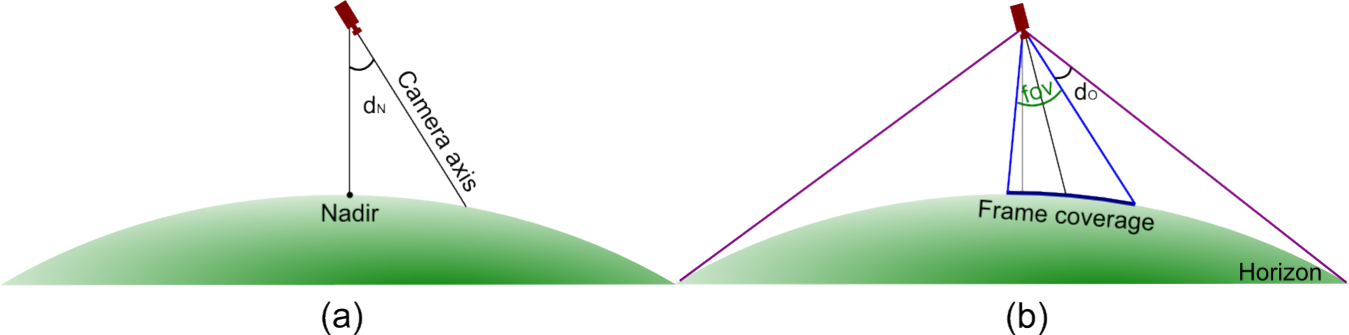
Filtering criteria: (**a**) angle between the camera optical axis and the line from camera optical center to nadir point; (**b**) angular difference between camera field of view and the cone covering the horizon circle

An optimal video sequence for building a mosaic should be taken with the camera pointing the nadir (*i.e*., vertically oriented) since the farthest is the deviation from the vertical axis, the highest is the perspective distortion affecting the frame. However, in real sequences, the camera will assume different orientations according to the needs of the operators and of the target application. Therefore, frames that are not suitable for building the mosaic should be discarded. Frame rejection is based on the following four criteria (Figure 5):

1. if the camera altitude over the ground is too low, the frame is discarded to crop away aircraft take-off and landing;
2. assuming a pinhole camera model, if the angular deviation *d_N_* between the camera optical axis and the line connecting the camera optical center and the nadir point is higher than a fixed threshold, the frame is discarded;
3. if the nadir point falls outside the frame, the frame is discarded;
4. if the minimum angular difference *d_O_* between the camera field of view and the cone covering the horizon circle from the camera optical center is lower than a fixed threshold, the frame is discarded to avoid including pieces of sky in the mosaic.

Another factor related to the choice of retaining or discarding an incoming frame is the degree of overlap between its orthoprojection and the current mosaic, which can be computed as soon as the ground position of the image corners are known. If the overlap percentage becomes too low, the amount of common information might hamper a correct visual alignment while, if it becomes too high, the redundancy burdens the computational time and is more likely to produce blurred areas in the mosaic. Maximum and minimum overlap thresholds have been heuristically fixed to, respectively, 75% and 10%.

### 5.2. Alignment

Orthoframe geo-referencing is not sufficiently precise to provide a seamless merging for creating the final mosaic and, therefore, it must be refined. To improve frames alignment, their visual content is exploited: since consecutive orthoframes partially overlap, it is possible to arrange their relative position so that their common parts match. In the following, this operation will be referred to as *visual alignment* to distinguish it from *geographic alignment*, defined by geo-registration data.

**Figure 6.**
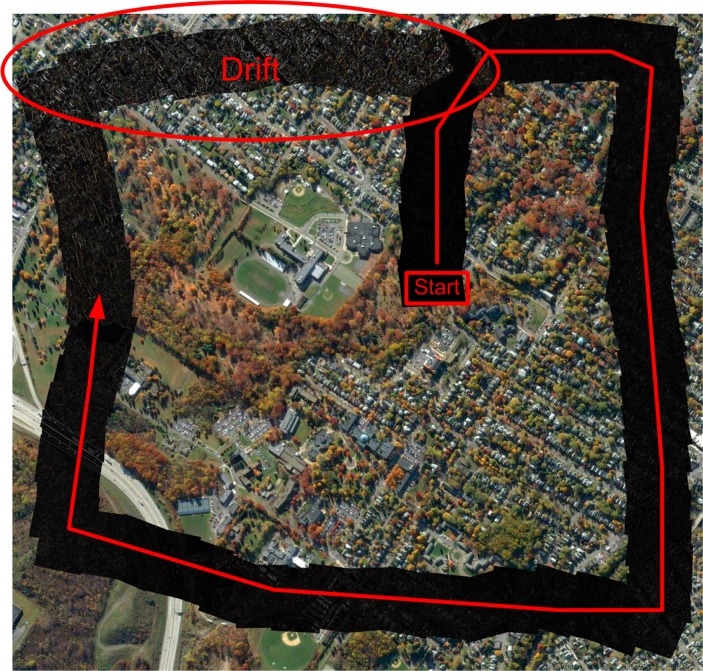
Difference between a reference ortho-photo and a mosaic strip showing the increasing drift in the final part.

Visual alignment allows for seamless mosaicking, but the process is intrinsically drifting, affecting the mosaic metric precision. An example can be seen in Figure 6, where a large ortho-photo has been used to generate a synthetic mosaic. A set of patches simulating an aerial image sequence, captured by an UAV following a certain path, have been extracted together with their (virtual) telemetry data. Figure 6 shows the difference between the reconstructed mosaic and the original image, where the grey levels of the common pixels are proportional to the distance in RGB space between the two images.

Geographic alignment, on the contrary, is less precise but unaffected by drift. Therefore, our answer to the drift problem was to use data from geographic alignment to periodically reset the output of the visual alignment if their difference in position or orientation was higher than a predefined threshold. Finally, we performed a global alignment and a subsequent finer orthoframe registration in order to reduce accumulated errors and consequent frame misalignments.

#### 5.2.1. Visual Alignment

Visual alignment is based on a set of image correspondences that are computed exploiting the *SIFT* algorithm [45]. SIFT detects distinctive local features in monochromatic images that are invariant to image scale and rotation and provide robust matching across a substantial range of affine transformations, 3D viewpoint changes, noise corrupted data and illumination changes. SIFT is a de-facto standard for image composition but its applications include as well object recognition, robotic mapping and navigation, video tracking and match moving. Since SIFT algorithm has a high computational cost, it has been ported to GPU, re-implementing it in OpenCL, in order to meet near real time execution.

Visual alignment between the current orthoframe *O_i_* and the previous valid orthoframe *O*_*i*−1_ is computed as follows. A set *f_i_* of SIFT descriptors is extracted from *O_i_* and correspondences between elements in *f_i_* and *f*_*i*−1_ are computed according to their similarities, defined by the Euclidean distance of the SIFT descriptors. Then, RANSAC algorithm [46] is used to discard outliers, obtaining a consistent subset of correspondences (Figure 8). Finally, the transformation matrix *W*_*i*,*i*−1_ aligning *O_i_* to *O*_*i*−1_, is computed by solving the non-linear least squares problem defined by the robust correspondences with a Levenberg-Marquadt algorithm [47].

Frames with poor visual contents may lead to small feature sets whose size is not sufficient to compute a reliable transformation matrix. In this case, the geographic alignment is again used instead of the visual one.

**Figure 7.**
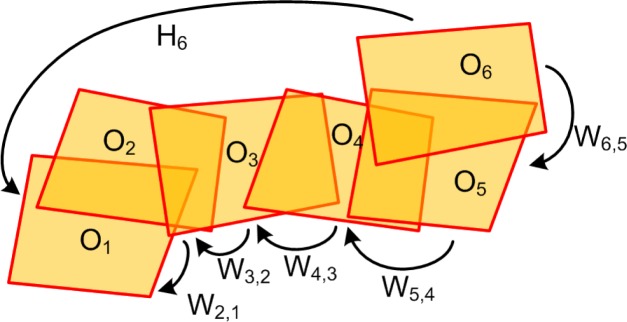
Computing the transformation matrix *H_i_* of an orthoframe as a chain product of the alignment matrices *W*_*i*,*i*−1_ between consecutive frames of the input sequence.

**Figure 8.**
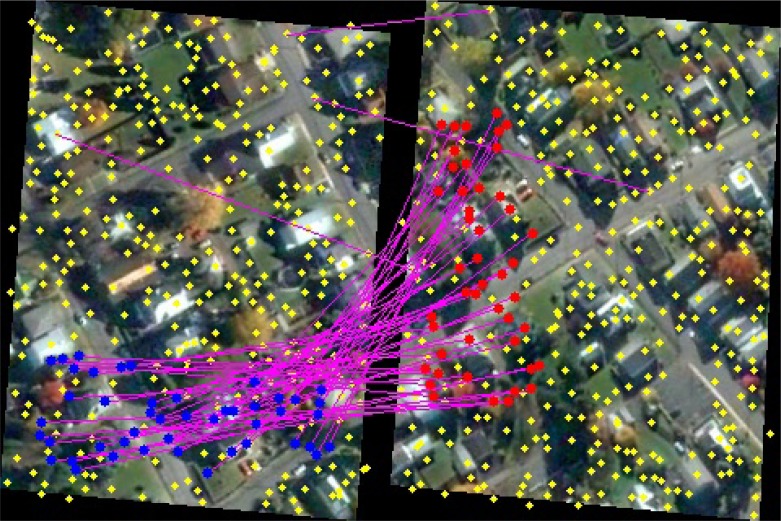
SIFT feature points extracted from two consecutive frames (yellow marks). Feature descriptors are matched, establishing correspondences (purple lines), which are then filtered through RANSAC obtaining a consistent subset (blue and red marks).

In order to build a single mosaic, all frames must be aligned w.r.t. a common reference system. It can be convenient to choose the coordinate system of the first frame of the sequence as the common one. Therefore, the transformation matrix *H_i_* aligning *O_i_* to *O*_1_ can be computed as a chain of matrix products (see Figure 7):

(1) $$H_{i}=W_{2,1}W_{3,2}\cdots W_{i-1,i-2}W_{i,i-1}=\prod_{l=2}^{i}W_{l,l-1}=H_{i-1}W_{i,i-1}$$

Summarizing, the outline of the orthoframe alignment algorithm is the following:

*G_i_* = geographic alignment matrix *G_i_* computed from geo-referencing data;
*f_i_* = {SIFT features extracted from *O_i_*};
match the features in *f_i_* and *f_i_*_−1_;
filter the correspondences with RANSAC algorithm;
**if***the number of robust correspondences is lower than a predefined threshold***then**
  *H_i_* = *G_i_*;
**else**
  evaluate *W_i,i_*_−1_ with Levenberg–Marquadt algorithm;
  *H_i_* = *H_i_*_−1_*W_i,i_*_−1_;
  **if***the rotation or translation differences between G_i_ and H_i_ are higher than a predefined*
  *threshold***then**
    *H_i_* = *G_i_*;
  **end**
**end**

#### 5.2.2. Global Optimization

The homographies computed in Equation (1) are obtained optimizing locally their parameters, since they consider only two consecutive frames. Therefore, in order to improve globally their precision and to reduce the accumulated errors, a bundle adjustment is applied. In this process, we try to optimize the *H_i_* parameters in order to minimize, again with a Levenberg–Marquadt algorithm, an objective function based on the squared sum of the differences between the set of transformed robust features of each image and their corresponding elements in all matching frames. Images are added one by one to the bundle adjuster according to the (decreasing) number of available correspondences with other orthoframes.

### 5.3. Stitching

Stitching is the second stage of the mosaicking algorithm. It is executed only once all frames have been received, orthorectified and aligned. Since the final mosaic can be extremely large, it is constructed as a grid of rectangular patches of fixed size. This requires to identify which frame pixels belong to each patch. The patches are squares of size *p_S_* = 1*,* 536 pixels and are aligned w.r.t. the global reference system. The single patches are built independently, by blending pixel values over overlapping areas of the incoming frames in order to achieve seamless smooth transitions.

**Figure 9.**
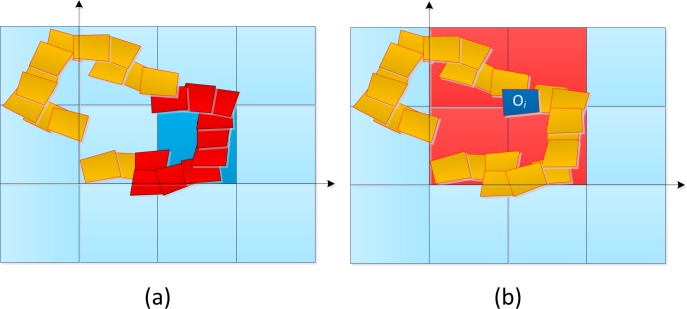
The overall mosaic is created as a grid of patches. In (**a**), the blue patch intersects all the red orthoframes; In (**b**), the current frame *O* intersects the red patches and, therefore, will be added to their reference lists.

The stitching algorithm is divided into two parts. First, the list of frames candidates to contribute to a patch is obtained by intersecting the convex-hull of each orthorectified frame *O_i_* with each patch. An example can be seen in Figure 9, showing in the red color the list of frames intersecting a patch (a) and the list of patches a frame will contribute to (b).

Then, each patch is created with a pixel-wise strategy: for each pixel of the current patch, the candidates contributing values are retrieved from the list of overlapping orthoframes. If more than a candidate is present, some technique must be adopted to calculate the final pixel value. This technique must take care of some issues:

- when there are more candidates for a patch pixel, the ones closer to their frame center, where orthorectification artifacts are less severe, are the most reliable;
- to avoid discontinuities in the final mosaic, some blending should be performed in areas where multiple frames overlap;
- blending introduces blur in the mosaic, so it should be used carefully and only when necessary. Furthermore, if the alignment is not correct and some visual features do not match in overlapping frames, ghost artifact’s will appear. So, even if overlapping areas can be very extended, blending inside them should be restricted to the region where it is necessary to prevent seams between frames.

**Figure 10.**
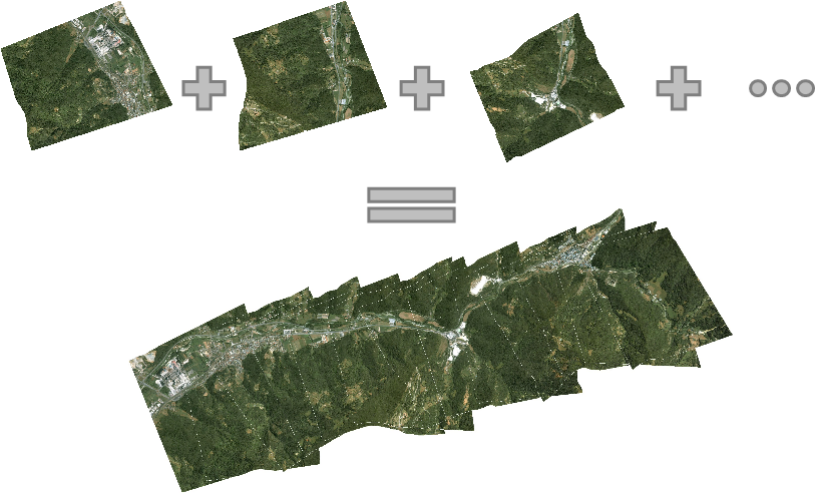
An example of the final mosaic obtained stitching several orthorectified images.

In our first prototypical implementation, we considered a simple weighted fusion of overlapping areas. Each patch pixel is assigned the blending of up to two values, taken from the candidate orthoframes having their centres closer to the current pixel. Blending weights are proportional to the inverse of the distances from the corresponding frame centres. An orthoframe mosaicking example is shown in Figure 10, where the border of each original orthoframe is represented by a dotted line to highlight their relative superimposition. However, as already stated, this technique, as any other blending technique, is affected by the problem of ghosting. As future work, we are planning to implement more complex fusion techniques, as the one proposed in [30], where an energy based local criterion is used to find an optimal seam-line. The energy based criterion tries to reduce the color information and the structural differences between the pixels to merge.

### 5.4. Performance Evaluation

The described algorithm has been implemented and tested on a set of aerial sequences acquired during the development of the UAV platforms in the SMAT-F1 project. All images were taken in the visible spectrum with on-board RGB cameras. Since the execution time depends on several factors, like image size, number of visual features, number of images handled by the bundle adjuster and so on, we computed an average value on different test sequences. The set of valid frames, those surviving the filtering described in Section 5.1.1, were divided into blocks of 500 elements and each of them was processed separately. For each frame, we recorded the time spent in ortho-rectifying the frame and computing its visual alignment. Then we summed up all these values with the time required by the bundle adjuster to optimize frame homographies. The average execution time for ten test sequences and frame size of 640 *×* 480 pixels was about 300 s. The resulting average frame processing time is 0.6 s, which can be considered a very positive result, not too far from a real-time execution.

## 6. Data Enrichment Using VGI

At the end of 2011, there were over 5.8 billion mobile-cellular subscriptions. Particularly, smart phones and tablets are already widely distributed and increasingly present year by year. These devices are often equipped with different accessories such as for example the global positioning system receiver (GPS), compass, Internet access, camera, microphone, Bluetooth module, *etc*. For this reason they can be considered as a multisensor platform in your pocket.

Thanks to these sensors, to broadband connections and to the Web 2.0 tools, a large number of individuals became able to create and share Volunteered Geographic Information (VGI) [48]. VGI is known as the spatial case of user-generated content phenomenon, that is, the phenomenon where Web users create content that is later integrated and made available through Web sites.

VGI is a phenomenon that has grown significantly in recent years and is still growing. Examples of sites implementing VGI in a Web 2.0 framework include: WikiMapia [49], which is an on-line editable map that combines Google Maps with a Wiki system that allows users to add notes to any place on Earth; Flickr [50], which collects geo-referenced photographs and allows the users to insert as a tag an image description; Google Map Maker [51], which is a service designed to expand the breadth of the service currently offered by Google Maps; Geonames [52], which is a geographical database containing millions of geographical names, formally categorized within a taxonomy. OpenStreetMap [53] is an open, collaborative project for building a map of the world. Each contributor develops a map using GPS tracking and individual contributions are assembled and reconciled into a single patchwork. Extensive metadata are incorporated, since each piece of the patchwork may have different levels of accuracy and may have been acquired at different dates.

VGI phenomenon has some clear benefits: the sources of information are distributed and dynamic and the cost of data collection is very low; VGI is constantly updated and expanded from providers that often produce very accurate data in a short period of time.

VGIs have also some disadvantages: The level of detail of the collected information is heterogeneous. This is strongly related to the availability of volunteers and has a direct impact on the completeness of the information. Often, VGI data are not controlled by a central organization responsible for it and this directly impacts on the quality and on the accuracy of the data. Furthermore, the integration of heterogeneous data and information content from multiple sources poses semantic interoperability issues.

As remarked by Michael Goodchild in his keynotes speech entitled *It’s About Time: The Temporal Dimension in VGI* held on February 2012 at GIS Week, in Redlands, California: “The most compelling case for VGI is during emergencies. Being the experts sometimes scarce on the ground, it’s important to turn to citizens who can contribute data through social media or other means. [..] We have seven billion intelligent observers on the planet.” Humans, equipped with some working subset of the five senses and with the intelligence to compile and interpret what they sense are intelligent synthesizer and interpreter of local information. One can see VGI as an effective use of this network, enabled by Web 2.0 and the technology of broadband communication [48].

### 6.1. VGI and Map Mash-Up

Today, almost all VGIs provide Application Programming Interfaces-API, set of technologies that enable Web sites to interact with each other by using REST [54], SOAP [55], JavaScript [56], RSS [57] and other Web technologies. APIs facilitate the development of Web mash-ups, *i.e*., applications that combine multiple sources of data and/or services to create new useful Web applications. Originally the term mash-up was used to describe the mixing or blending together of musical tracks, but now the term refers to applications that combine data from different sources into new Web services in order to produce new meaningful information. The availability of APIs has fostered the concept of a “mash-up” as the ideal presentation vehicle for VGI by providing a geographical backdrop [58]. APIs have spurred the growth of the Geospatial Web and represent spatially aware online communities and new ways of enabling communities to share information from the bottom up [59]. Map “mash-ups”, through the use of frequently updated data from multiple sources, allow seeing micro-behavior spatio-temporally. As such, crisis map mash-ups are emerging as interesting artifacts in the practical work of reporting on, assisting in, and managing emergencies [60].

A map mash-up combines at least one map data source or service with added information, often geo-referenced to the map data, to create a new map [61]. When data providers do not expose APIs, mash-ups are created using a technique known as screen scraping [62]. Screen scraping denotes the automatic extraction of information from the provider’s Web pages that are originally intended for human consumption.

As already said, API are typical mash-ups content providers. As regards the hosting site, which is where the logic of mash-up resides, it can be implemented using server-side content generation technologies (e.g., servlet, PHP) or directly within the client’s browser through client-side scripting or applets. The client’s Web browser is where the application is rendered. The browser is used to graphically display the mash-up and to allow the user to interact with the mash-up Web site. The client-side logic is often the combination of code directly embedded in the mash-up’s Web pages as well as scripting API libraries or applets (furnished by the content providers) referenced by these Web pages. Mash-ups using this approach can be termed Rich Internet Applications (RIAs) [63], meaning that they are very oriented towards the interactive user-experience [64]. Very often mash-ups use a combination of both server and client-side logic with the overall effect of enabling the user to make smaller requests for information to the client-side, reducing the need for the remote Web site to process ever single request remotely

### 6.2. OpenStreetMap

OpenStreetMap (OSM) is a collaborative project to create a free editable map of the World. It follows the same peer production model that has been used in many other well-known initiatives such as Wikipedia. The set of map data are free to use, editable, and licensed under new copyright schemes. Users can produce data using handheld GPS devices or Yahoo Imagery or other free map sources. The OSM Wiki page provides detailed instructions for beginners on how to collect and contribute data to the Web site. Users can actively add data to the map using different types of editors.

OSM maps are made up of three basic elements: nodes, ways and relations. They correspond to the entities that are stored in the OSM database. Each element may have an arbitrary number of properties (tags) that are key-value pairs, such as *highway=primary*. A node is the basic element of the OSM scheme and represents a map feature or a standalone entity. It must have at least one tag and consist of the coordinates (Lat/Lon), the user name that provided the data and a time-stamp. Linear entities called ways are defined by reference to an ordered list of points. A relation can group other elements together, nodes, ways, and even other relations. Along with the geographical coordinates of the features in the OSM database, the feature attributes are recorded for each node and way as semicolon-separated key=value pairs (for example, type=pub; name=The Bull). This information organization is the tagging schema that is provided to the system users. This information organization schema is increasingly being developed into a complex taxonomy of real-world feature classes and objects. It corresponds to the core part of the OSM initiative and is community-driven. Any member of the community can contribute to an update the schema by proposing new key=value pairs [65].

OpenStreetMap provides API to end users. For this reason it can be used as a data source for different types of mash-ups. In the following section we describe the mash-up architecture we developed for the Second Level Exploitation function and how we exploited OpenStreetMap data.

### 6.3. Mash-Up Architecture

**Figure 11.**
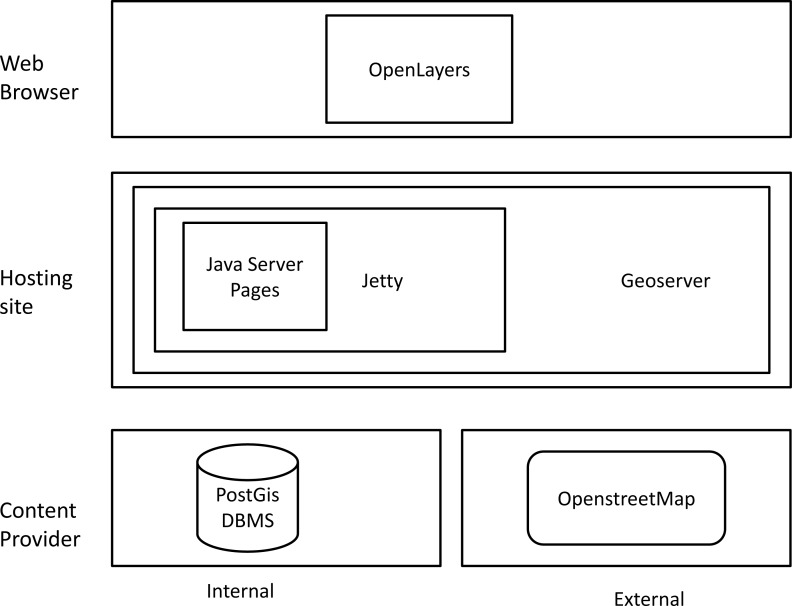
The mash-up software architecture for the SMAT- F1 Second Level Exploitation.

As shown in Figure 11, the mash-up architecture followed the three tier general architectural model described before. In the following we describe the main technological sources used for each tier.

**Content Provider.** The content provider included an external resource represented by the OpenStreetMap data and an internal resource represented by the data stored in the system database (the DBMS PostgreSQL and PostGIS).

OpenStreetMap data were exploited via APIs calls. Indeed, APIs enable access to the OSM database, allowing user authentication, addition, update and deletion of geographical features. There are API calls to retrieve map data by bounding box, to create/retrieve change-set, to add, delete and update the three basic elements: node, way and relation. Each of them returns or expects the data for the elements in OSM (XML) format with UTF-8 character encoding. The REST requests take the form of HTTP GET, PUT, POST, and DELETE messages. Requests to modify the database are authorized using HTTP Basic Authorization or OAuth. Read requests do not require authorization.

In the implementation of the mash-up the API request that allows the retrieval of map data by bounding box was used. This particular API request allows to specify a bounding box, identified by the coordinates of the vertices of a square region. Vertices are defined in terms of their latitude and longitude. The API call retrieves:

- All the nodes that are inside the bounding box and any relation that makes reference to them.
- All the ways that refers to at least one node inside the bounding box; any relations that refer to the ways; any nodes outside the bounding box referred by the ways.
- All the relations that refer to one of the nodes or ways included in the result set due to the above rules.

The internal source of content was represented by the data stored in the system DBMS: PostgreSQL [66]. PostgreSQL is a powerful open source DBMS. With its spatial extension PostGIS [67], that adds support for geographic objects to the PostgreSQL database, it allowed archival, retrieval and management of all the data received, processed and generated in the SMAT-F1 project. In addition, it provided the capability for cataloguing images and data in terms of spatial data indexing.

**Mash-Up Logical Component.** In the hosting site, GeoServer [68] was used. GeoServer is a Java based, platform independent, server side software. It allows users to view, edit and publish geo-spatial data. GeoServer reads a variety of data formats and through standard protocols it produces KML, GML, Shapefile, GeoRSS, PDF, GeoJSON, JPEG, GIF, SVG, PNG and more. GeoServer is the reference implementation of the Web Feature Service (WFS) [69] and the Web Coverage Service (WCS) [70] standards of the Open Geospatial Consortium (OGC). It provides a high performance, certified compliant support to the Web Map Service standard (WMS) [71]. GeoServer connects to a wide variety of existing public geo-spatial data sources over the Web such as Google Earth, NASA’s World Wind, Yahoo! Maps plus many others [72]. GeoServer uses Jetty as a servlets container. Jetty is an open source middle-ware that provides a HTTP server and Servlet container capable of serving static and dynamic content either from a standalone or embedded instantiations. Jetty can handle Java Servlets [73], JavaServer Pages (JSP) [74] technologies and traditional static Web pages. JSP is a technology designed for the development of Web based applications and is based on the Servlet technology. The main distinction between a JSP and a Servlet is that a JSP is an HTML page with Java code embedded within HTML tags. Instead, a servlet is constituted by Java code that generates the Web page. On initial access the JSP is converted into servlet source code and then compiled and executed.

**Mash-Up Web Browser.** On the user layer of the architecture there was the client Web browser and OpenLayers [75]. OpenLayers is an open source JavaScript library for displaying map data in Web browsers. OpenLayers provides a JavaScript API for building rich Web-based geographical applications. Data can be combined from a number of sources without requiring any server side processing since the layers can be assembled and rendered on the client. Client side programming includes panning and zooming of maps, client-side tiling, markers, pop up windows, various navigation components, keyboard commands, an event handling mechanism and client server communications. OpenLayers allows overlying markers, icons and cartography on different base maps. It can return projection information and it provides tools to help programmers to re-project data on the client side. OpenLayers can act as a Web Client for OGC Web services (WFS-T, WMS, and WCS), commercial services such as Google Maps, MSN Virtual Earth, ESRI products, open source initiatives or de facto standards such as Geographically Encoded Objects for RSS feeds (GeoRSS) [76].

**Figure 12.**
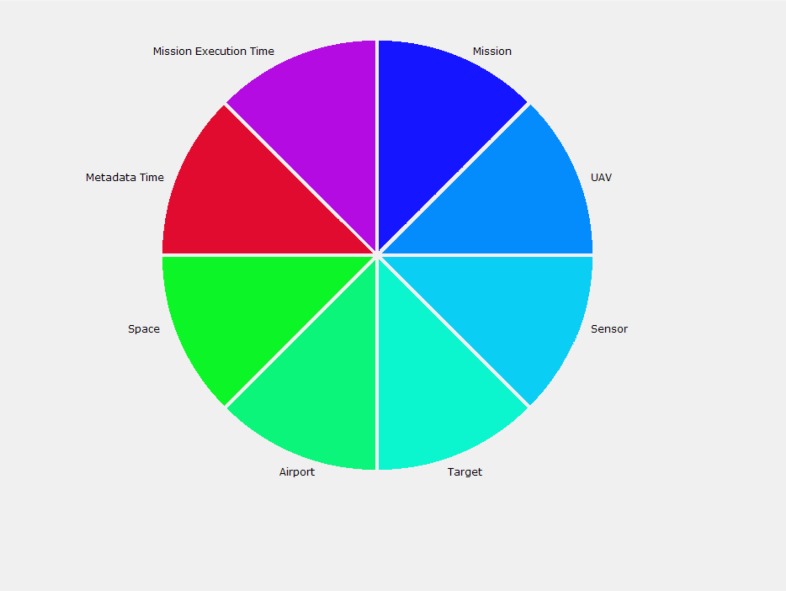
Graphical user interface for the retrieval of spatial objects and the associated metadata.

**Data Enrichment and Mash-Up Results.** The SS&C operator queried the relational database through the Web browser. In particular, the user’s requests searched for metadata related to some spatial objects. Often the spatial objects have been already involved in some mission facts, according to the multi-dimensional data model developed for the project. Thus, the user query searches for spatial objects of interest according to their description in the data model. The mission’s facts in the data model were described by the following independent dimensions: UAV, sensor, target, airport, mission execution time, metadata time, space (coordinates). The graphical user interface (see Figure 12) shows each of the dimensions with a colored sector. The user, by clicking the sectors, entered in a new window that allowed the user to specify additional constraints on the values of that dimension. Consequently, the system performed the user query on the mission data satisfying the constraints and the mash-up logic translated the user query into a set of SQL queries for the system database.

The first type of SQL query retrieved the spatial objects satisfying the constraints and returned the spatial coordinates of the corresponding locations. These spatial coordinates are necessary to perform a following Web service request to OpenStreetMap that is used to retrieve the available metadata on those spatial locations. The second query returned the metadata already stored in the local database for the same spatial objects.

The output produced by the system was the metadata associated to the spatial objects satisfying all the constraints imposed on the dimensions. The answer of OSM is constituted by an XML file for each spatial object. Each file was parsed and the identified annotation tags were presented in the output page, grouped by category and ordered by time. The system checks the annotations and performs some actions of data cleaning.

**Figure 13.**
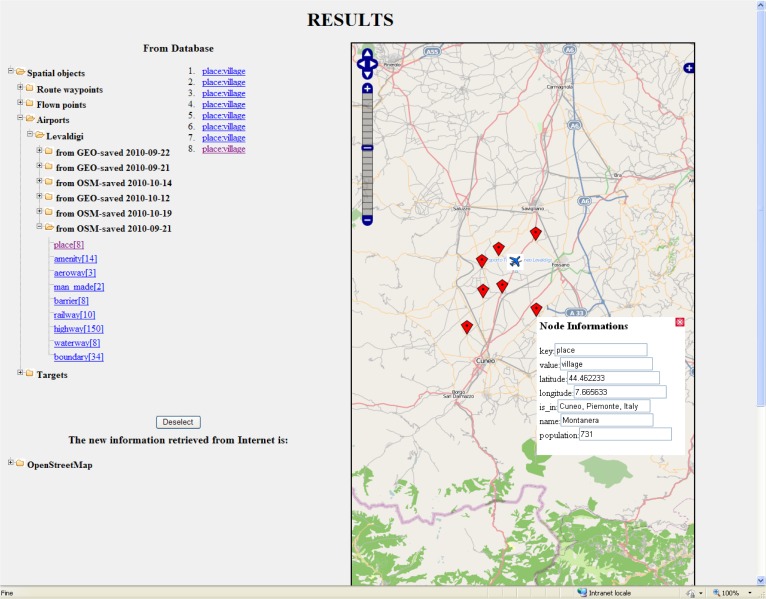
SMAT-F1 Interactive map with annotations.

Figure 13 shows an example of the output page produced by the enrichment function. The example shows the populated sites surrounding the airport of a surveillance mission (in Torino, Italy). The left-hand side of the window displays the annotations in a tree-like arrangement that helps the user to browse the annotations for the spatial objects of the query and orders them by the temporal reference. Each annotation on the left is geo-referenced by an icon in the map on the right.

## 7. Experiments and Results

In this section, we present some experiments on the ability of human sensors to provide useful information on a spatial area. The experiments have the purpose to deploy the information provided by the human sensors for the extraction of the characteristics that are specific of a spatial area. These specific characteristics are automatically selected by system so that they allow to perform a sharp distinction between the given area and the surrounding ones.

### 7.1. Statistical Filter on Annotations

Observing that in big metropolitan areas the annotations retrieved by the enrichment function were too abundant, with the risks of making the operator lost in the volume of suggested metadata, we decided to apply a filter to the retrieved tags. In addition, we noticed that some of the tags could not be relevant or could be the result of a mistake with a misleading effect similar to the superimposition of noise on the valuable information. In order to eliminate the noisy effects and validate the users’ annotations, we applied a filter that consisted in the extraction of the tags that appeared to be significant by a statistical validation method. The statistical method compared the frequency of occurrence of each tag category encountered in a given area, with the distribution of the frequencies of the same tag category in the surrounding geographical areas. Using the filter, we reduced the number of suggested tags and we were able to identify the typical features of an area as those ones that distinguish the given geographical area from the nearby regions.

#### 7.1.1. Statistical Method

Given an area where we wanted to apply the statistical filter, we built a regular grid composed of a total of 49 cells, surrounding the area.

**Figure 14.**
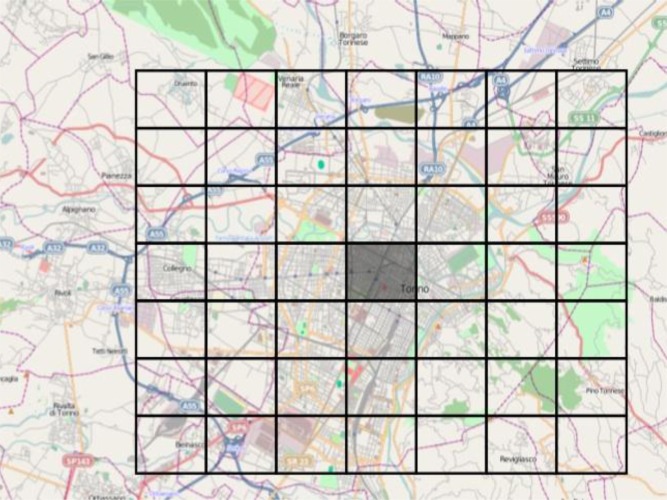
The area of interest with the surrounding grid cells.

All the cells of the grid had equal surface area of the central target cell. Thus, in any cell, each tag category had the same probability to appear (see Figure 14). The aim was to monitor the frequency of occurrence of each feature in the central cell of the grid and compare it with the frequency of the same feature in the neighbouring cells. (The features were denoted by the *key* attribute in each *key=value* OSM element description).

We used the statistical test on the mean [77], making a hypothesis test on the frequency of occurrence of each feature. We make the hypothesis that, given the spatial neighbourhood of the cells of the grid, all cells have the same law of feature distribution. In particular, the selection of 49 cells allowed us to apply the law of large numbers: if the sample is constituted by a sufficiently large number of cases (at least 30–40 cases), the statistics on the sample observations are distributed according to the normal distribution. In this case, each observation is constituted by the frequency with which a certain feature occurs in one of the cells of the grid.

More in detail, for each feature in the central cell, we computed its frequency in the central cell and its frequency in the neighbouring cells of the grid, central cell excluded. The neighbouring cells constituted the sample for the statistical test. We performed a standard test on the frequency of observation keeping the confidence level at the 99%. In the statistical test, a feature was statistically significant only if its occurrence frequency *f* in the central cell was such that *f > μ* + 3*σ* where *μ* and *σ* were the mean and the standard deviation computed from the frequency distribution of the feature in the neighbourhood cells.

**Figure 15.**
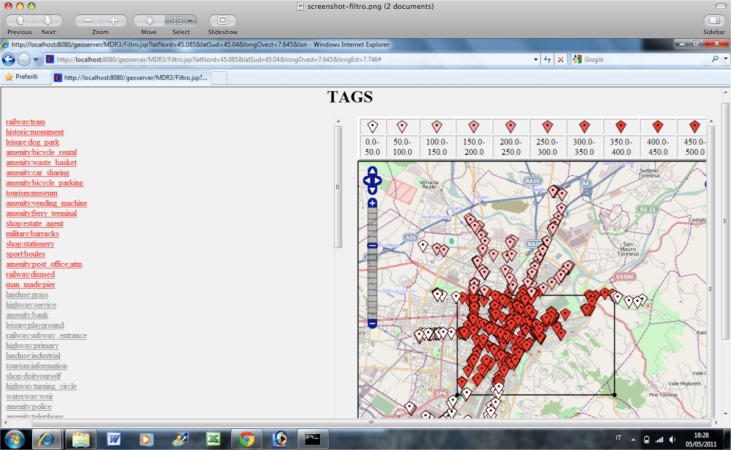
Filtered annotations (on bus-stops) are listed in red and appear in the map with a color that depends on their frequency.

In this way, the right tail of the normal distribution was examined in order to find if the frequency of the tag category in the central cell was an outlier. The frequencies of the tag categories in the given area that were outliers were highlighted as interesting ones. In fact, the tag categories expected to be selected by the filter process were: (i) the categories on which the majority of the users agreed (they were the most frequent ones) and that were not the result of some isolated cases; (ii) the tags that discriminated the area from the surroundings and thus characterized the area. At the end of the procedure, only the features that pass the filter are presented to the user as statistical significant features for that geographical area. Figure 15 shows a screenshot of the system with the result of the statistical filter on the annotations (tags).

The selected tags are shown in the list in red and are correctly placed in the map with a pin whose color depends on its frequency in the cell of the grid. In this way, the user immediately perceives both the spatial location of the annotation category and an aggregated property that refers to its frequency in the area.

#### 7.1.2. Experimental Results

Experiments on map characterization by metadata were run on an Intel dual core i3 330-m processor, with a 2.13 GHz clock. It can run up to 4 threads, with a 512 KB cache for the first core and a 3 MB cache for the second core. The RAM is DDR3 with 4 GB (at 1.066 MHz). Hard disk is 320 GB large, of SATA type and has a speed of 5400 rpm. Operative System is Windows 7 Home Premium, 64 bit. The system runs with a Web browser Internet Explorer 8.

In Table 1 we report the results obtained by an experimental study on a set of 90 spatial areas having the same radius (1 km), but chosen differently, as follows:

1. 30 metropolitan areas, with a high density of tags;
2. 30 scarcely populated areas;
3. 30 areas chosen randomly.

We report the tags source (OpenStreetMap or GeoNames, which is another open collaborative project with metadata on named locations coming from Wikipedia), the target object type monitored by the mission (airport or target), the average file size (over the 30 maps of the same type), the average time for the file download, the parsing time of the XML files and the tag insertion in the internal hash table, in preparation for the visualization, and finally, the average rendering time for the visualization of an object and all its annotations. Query compilation time (6–7 ms) is constant and does not depend on the amount of tags. Therefore it is not in Table 1. Compilation and visualization times (on average, 37 ms are needed for all the annotations of an object) are negligible with respect to the other times: the file download times (around 1800 ms on average) and the XML parsing times (around 120 ms on average). Anyway, even the higher download times are acceptable, considering that they are necessary to obtain hundreds of tags. Finally, experiments on the map statistical characterization confirm its utility. In random maps no significant tags are found, as expected. In metropolitan areas and even in scarcely populated areas, like Everest, significant tags are found.

In this paper we performed actually an evaluation from the viewpoint of the computational performances. We agree with the Web and the LinkedData communities that the user-generated content could be evaluated also from other perspectives. In fact it is known that at the current status, the user-generated content on the geographical domain does not cover all the areas uniformly, and that the quality of the metadata could still be improved. But it is also true that the quality is reported by Goodchild [38] to be rapidly improving thanks also to the many projects on LinkedData, such as the geoWeb/Silk [78] that aim to eliminate discrepancies in the concept representation. This is the matter of the future research in the SMAT project and the improvement of the research as reported also in the conclusions.

## 8. Conclusions

This paper summarizes our contribution to the SMAT-F1 project, a geo-spatial project that aims to collect data by payload sensors mounted on a flood of UAV for the territorial monitoring and protection. We described the SOA architecture of the system and the functionalities of the two software components that implement the second level exploitation function: aerial image mosaicking and the cartography enrichment function by metadata provided by human sensors. We showed that human sensors can be exploited to characterize a spatial area. We run a statistical test that filters the users provided annotation categories such that they can distinguish sharply the given spatial area by the surrounding ones. Furthermore, the time performances are acceptable and show that human sensors are a feasible solution to enrich spatial maps. Further work on human sensors regards the improvement of metadata quality. In fact, when metadata come from different social networks, such as GeoNames and OpenStreetMaps, the same named entity could be tagged in different ways by different users. This is a well-known problem since the integration of information systems with heterogeneous schema, and it is encountered again in the semantic web where different ontologies are used in the information sources to represent users’ tags and metadata. We plan to adopt machine learning technology (such as document classifiers and document clustering) to improve the quality of the tag categories, to reduce the amount of record duplications and to apply specific semantic representation languages (like RDF) and software solutions (like Silk [78]) for term cleaning and normalization.

## Figures and Tables

**Table 1.** Number of tags, XML file size, download times and parsing times for 90 areas of different types.

**Area type**	**Tag source**	**Object type**	**Avg n. of tags**	**Avg XML file size (bytes)**	**Avg download time (ms)**	**Avg parsing time (ms)**	**Avg rendering time per obj**
30 metropolitan areas	OSM	Airport	85.76	495,868.03	5,691.86	212.30	36.72
		Target	134.56	466,990.96	6,464.16	328.13	44.75
	GeoNames	Airport	10	10,172.30	1,412.23	94.80	32.37
		Target	6.03	5,727.26	728.43	78.93	27.57
30 scarcely populated areas	OSM	Airport	15	66,595.60	1,529.63	166.20	37.65
		Target	10.46	82,075.70	1,011.53	191.23	30.25
	GeoNames	Airport	0.40	397.36	1,117.16	37.43	36.50
		Target	0.26	239.06	245.70	47.33	42.35
30 random areas	OSM	Airport	4.16	100,996.30	1,443.86	171.10	37.28
		Target	4.73	96,420.70	1,024.30	149.10	38.75
	GeoNames	Airport	0	69	1,120.86	37.00	–
		Target	0.03	89.93	274.16	40.06	48.00
